# The Emerging Role of Oyster Mushrooms as a Functional Food for Complementary Cancer Therapy

**DOI:** 10.3390/foods14010128

**Published:** 2025-01-04

**Authors:** Priya Lakshmi Sreedharan, Malu Kishorkumar, Elke Gabriel Neumann, Shyam S. Kurup

**Affiliations:** Integrative Agriculture Department, College of Agriculture and Veterinary Medicine, UAE University, Al Ain P.O. Box 15551, United Arab Emirates; priya_s@uaeu.ac.ae (P.L.S.);

**Keywords:** bioactive compounds, natural antioxidants, functional foods, nutraceuticals, edible mushrooms

## Abstract

The importance of functional food’s role in human nutrition as well as in the prevention of diseases, especially the treatment of chronic diseases like cancer, is an innovative field of research. Based on the studies regarding the antioxidant potential of oyster mushroom extract, it is evident that it has anticancer properties. The current article reviews the health benefits of edible oyster-mushroom-derived bioactive compounds, and how they specifically activate or regulate the immune system by affecting the maturation, differentiation, and proliferation of immune cells, thereby inhibiting cancer cell metastasis and growth. Mushrooms show anticancer potential by regulating a single molecule of a specific signaling pathway or by having multiple targets in the same or different signaling pathways. In addition, the prebiotic effects of mushrooms could enhance quality of life during and after cancer therapy by recovering the intestinal microbiota. More clinical research on oyster mushrooms needs to be conducted, and future studies should investigate the preventive aspects, which aid in reducing the rate of cancer occurrence, and the positive impact in cancer patients to prove that oyster mushrooms are preventive as a functional food as well as a curing dietary supplement for cancer patients.

## 1. Introduction

Cancer is a diverse class of diseases, with the abnormal growth of cells being the second cause of death globally, the first being cardiovascular diseases. With an estimated 9.9 million deaths due to cancer in 2020, the constant search for cancer remedies has intensified research about bioactive compounds that can either prevent cancer or be used to treat it, hence the increasing interest in natural products originating from food. It is important to note that, to achieve relief from cancer-associated health problems and to reduce the side effects of chemotherapy, there is an increasing demand for anticancer functional foods among cancer patients [1]. Of these, edible mushrooms have received attention for their nutritional qualities as well as health benefits, particularly oyster mushrooms that belong to the species *Pleurotus ostreatus* [2]. Oyster mushrooms are not only highly valuable from a gastronomical point of view but also from the point of view of bioactive compounds, which have various pharmacological activities. It has been shown that these mushrooms have various kinds of antioxidants, phenolic compounds, flavonoids, and polysaccharides, which play an important role in protection against oxidative stress, which is an important cancer risk factor [3]. The presence of these antioxidants indicates that oyster mushrooms may assist in the protection of the body’s cells from damage and, thus, reduce the cancer cells through the scavenging of free radicals [4]. Beyond their dietary essentials, understanding the health benefits of mushrooms in the prevention of cancer will pave the way for novel steps in complementary cancer therapy as well as a reduction in the side effects of current therapy methods.

There is a growing interest in employing whole-food-based strategies to prevent chronic diseases, owing to the potential synergistic interactions among various bioactive components found within whole foods. For many decades, mushrooms have been consumed as food, and recent studies have proved that their major bioactive components can be utilized in making dietary supplements to improve the quality of human life. Oyster mushrooms are one of the most commercially important macro fungi due to their unique taste, nutritional qualities, and pharmacological importance, and they belong to the class Agaricomycetes and family Pleurotaceae. Furthermore, due to their low substrate specificity, they can be easily grown in several types of agricultural biowaste, which make them one of the most important commercial mushrooms globally [5]. Beyond their nutritional value, *Pleurotus ostreatus* has been established to exert remarkable growth inhibitory effects on several cancer-derived cell lines, such as breast, colon, and certain forms of liver cancer [6]. These effects, as well as the products of apoptosis in cancer cells, and antiangiogenic action, which is the suppression of the formation of new blood vessels on which the tumor depends for its growth and development, are reported in studies at various stages of the cell cycle [7]. Additionally, there has been an emphasis on the immunomodulatory perspective of these mushrooms, especially regarding the oyster mushroom species. In general, substances present in these mushrooms can stimulate macrophages and natural killer cells, thus inhibiting the tumor promotion and metastatic potential of the cancer cells [8]. In addition to their antioxidant potential and their ability to trigger immunity at the same time, oyster mushrooms may be viewed as having the potential for adjuvant therapies for several types of cancers [9]. However, these outcomes prompt the need for serious and more extensive investigations regarding the specific molecular mechanism of bioactive compounds present in the fruiting body as well as in the mycelium, specifically regarding how they exert such impacts on the human body.

Moreover, the awareness of the special health-promoting qualities of oyster mushrooms might open new methods of cancer treatment through a mushroom-based, balanced diet. In general, with an increase in the prevalence of cancer in the global population, it is prudent to consider nutraceuticals from natural sources like oyster mushrooms for inclusion in dietary prescriptions to improve the survival rates and to enhance the quality of life of human beings who have these ailments. More clinical studies must be conducted regarding oyster-mushroom-based dietary supplements, and the purpose of this review is to stimulate the scientific community toward further investigation into the prospect of oyster mushrooms as a rich supply of anticancer compounds. The anticancer compounds derived from mushrooms suppress cancer and improve the quality of life of cancer patients [10]. Nevertheless, more future studies are needed to understand the positive effects of oyster mushroom consumption in various cancer stages. More future research should be conducted in the form of longitudinal clinical trials with higher numbers of participants to determine the usefulness of oyster mushrooms as a complementary cancer treatment. These studies should try to determine the appropriate doses, as well as possible combinations with conventional chemotherapy or radiotherapy, and the effects of fresh, dried, or extracts of oyster mushrooms in cancer patients. Thus, in this review article, the current available knowledge of oyster mushrooms is discussed, specifically relating to its perspective as a functional food and source for bioactive compounds that induce such effects. The underlying processes, any further research needed, and the clinical prospects regarding the importance of how a mushroom-based diet can be utilized for complementary cancer treatment to reduce the side effects of chemotherapy are also discussed.

## 2. Bioactive Compounds in Oyster Mushrooms

**Figure 1:**
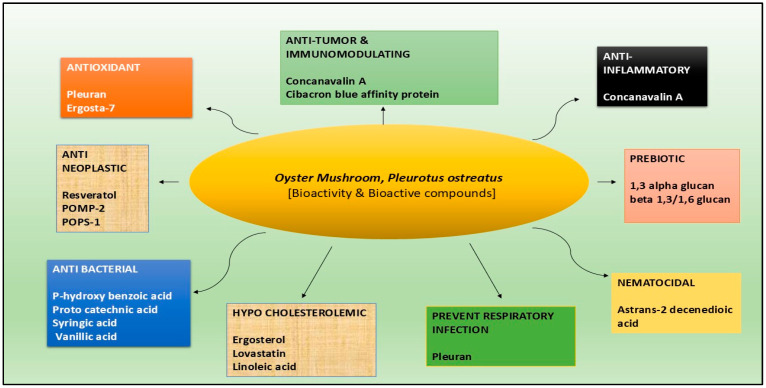
The bioactive compounds in the oyster mushroom *Pleurotus ostreatus* have several health benefits, making them vital in the functional food category; specifically, antioxidant, antineoplastic, antitumor, and immunomodulating activities make them an ideal adjuvant for cancer therapy.

The species *Pleurotus ostreatus* is often referred to as oyster mushroom and has been identified to have very high antioxidant activity, which is the result of some bioactive compounds present within the mushroom. Other oyster mushrooms, namely *Pleurotus djamor* (pink oyster) and *Pleurotus citrinopileatus* (yellow oyster), are also outstanding sources of bioactive compounds and can provide many health benefits. These mushrooms most commonly contain high molecular weight as well as low molecular weight bioactive compounds that provide these medicinal and nutritional values [11]. Most of the compounds in the oyster mushrooms have high molecular weight, which mainly comprises polysaccharides, like the β-glucans that are often thought to possess immunomodulatory as well as antineoplastic characteristics. Specific types of these polysaccharides can promote the immune response and increase the activity of macrophages and natural killer cells, which improve the body’s defense against cancer [12]. Furthermore, polysaccharides from *Pleurotus* species have an antioxidant effect, which can buffer oxidative stress that relates to a variety of diseases, including tumor formation and development [13]. However, oyster mushrooms also have low molecular weight compounds as well, and these are phenolic compounds, flavonoids and terpenoids. Some of these compounds are reported to possess antioxidant, anti-inflammatory, and anticancer properties. For instance, Chopra et al. have pointed out that phenolic compounds are capable of neutralizing free radicals, hence preventing any oxidative damage within the cells or tissues [14]. Therefore, oyster mushrooms, especially *Pleurotus ostreatus*, are a potential source of bioactive compounds, such as polysaccharides, phenolic compounds, and flavonoids, with antioxidant and anticancer effects through actions like preventing free radical formation, antioxidant production, and the modulation of the immune system. Oyster mushrooms’ nutritional profile comprises all the essential amino acids, high-quality protein, and the presence of bioactive compounds with antioxidant activity that makes them an ideal source for myco-pharmaceutical as well as for nutraceutical production [4]. Moreover, nutritional qualities, like low calorific value, referring to less carbohydrate and fat, as well as low sodium content and antilipidemic potential with the presence of riboflavin, selenium, potassium, niacin, proteins, fiber etc., in a balanced proportion make oyster mushrooms a perfect ingredient for formulating functional food for patients and high-risk individuals to improve their quality of life [15,16]. The important health-beneficial bioactive properties of oyster mushrooms are illustrated in Figure 1.

## 3. Health Benefits, Antioxidant Properties, and Anticancer Activities of Oyster Mushrooms

As illustrated in Figure 1, several health benefits of bioactive compounds purified from *Pleurotus ostreatus* have been reported and described in the literature. Besides, according to several other studies, it was demonstrated that the bioactive compounds present in oyster mushrooms have multiple valuable functions on health, particularly, these compounds have the capabilities of regulating diabetes, preventing atherosclerosis, and curtailing inflammation, which are the main causes of chronic diseases [17,18]. In addition, polysaccharides have immunostimulant features that can improve the immune system of the body, making oyster mushrooms a dietary intervention for improving immunity and reducing diseases [19]. Because the extracts possess a high concentration of bioactive compounds, oyster mushrooms are being used more and more in dietary supplements, as functional foods, and even in the cosmetics industry. These products exploit the health-promoting ability of mushrooms, giving consumers a natural approach for improving their health [20,21]. Adding these mushrooms to various food products enhances nutritional quality, with the bonus of several health benefits [22,23]. It is evident from all the publicized reports that oyster mushrooms are a valuable source of bioactive compounds that offer a multitude of health benefits, and their high molecular weight polysaccharides and low molecular weight phenolic compounds contribute to their immunomodulatory, antioxidant, and anticancer properties. As research continues to uncover the potential of these mushrooms, they are likely to play an increasingly important role in the development of functional foods and dietary supplements, aimed at promoting health besides preventing diseases, as they have the highest antioxidant, antiviral, and anticancer activities [24]. Among the various kinds of oyster mushrooms, *Pleurotus ostreatus* has become a subject of interest in cancer research because of its potential for anticancer effects. Thus, many works have confirmed the antitumor activity of the extracts from the edible mushroom described above, which affects several types of cancer cells, including those of breast cancer, colon cancer, and liver cancer [25]. The mechanisms through which these anticancer effects are produced are complex and varied. Thus, it can be concluded that the use of *Pleurotus ostreatus* as an antioxidant is highly effective due to the presence of bioactive compounds that can scavenge free radicals, increase the activity of antioxidant enzymes, and inhibit the generation of free radicals. These mechanisms taken together enable the mushroom to combat oxidative stress and enhance the general health of the body. Considering these facts, the possibility of using *P. ostreatus* in the diet is promising to prevent diseases allied with oxidative stress, including cardiovascular diseases, diabetes, and cancer. More studies are required to establish the total potential of its antioxidant properties and possible consequences on the health of an individual. Since the morbidity of cancer is increasing, suggesting oyster mushrooms as a part of the diet can be an inexpensive and practical way to help cancer patients as well as to prevent cancer in healthy individuals as “prevention is better than cure”.

### 3.1. Antioxidant Properties of Oyster Mushrooms

Antioxidants are known as “free radical scavengers”, as their function is to scavenge or neutralize free radicals, thus preventing the damage caused by oxidation. Free radicals are unstable atoms that can damage cellular DNA. Reactive oxygen species (ROS), which constitute a subset of free radicals, contain oxygen, which modifies cellular macromolecules, especially genomic DNA, which ultimately results in mutational changes leading to uncontrolled cell proliferation or cancer. Therefore, antioxidants may play an important role in the prevention of cancer as well as in cancer therapy, as they can reduce oxidative stress by scavenging ROS, thus preventing the DNA damage caused by oxidation [26,27]. In general, several species of oyster mushrooms, especially *Pleurotus ostreatus*, are well known to have antioxidant activities. As reported by research studies, these mushrooms are rich sources of bioactive compounds that display antioxidant potential, which may serve as the basis for the therapeutic effects [28]. Of these compounds, phenolic compounds are worth mentioning here. *Pleurotus ostreatus* contains a considerable amount of phenolics, such as gallic acid, chlorogenic acid, and naringenin, whose major function is the elimination of free radicals and the moderation of the degree of oxidation. Furthermore, besides phenolic compounds, *Pleurotus ostreatus* also possesses amino acid, namely ergothioneine, with outstanding antioxidant activity; it is involved in the protection of damaged cells and can help to reduce the prevalence of various diseases. All these bioactive compounds together account for the total antioxidant capacity of oyster mushrooms [12,29]. Altogether, there are several methodologies to analyze the antioxidant potential of *Pleurotus ostreatus*, like the 2,2-Diphenyl-1-picryl hydrazyl (DPPH) radical scavenging activity and the ferric reducing antioxidant power (FRAP) assay. According to the study conducted by Bakir and his research group, it was reported that the antioxidant potential of *Pleurotus ostreatus* is influenced by the temperature of the storage environment, emphasizing the importance of the effect of temperature for maintaining the antioxidant activity of the extract. The study also showed considerable antioxidant activity in the DPPH radical scavenging assay of the extracts [30]. Another study by Arbaayah and Umi Kalsom further focused on the differences in the antioxidant activities of different species of oyster mushrooms, including *Pleurotus ostreatus.* The results showed that *Pleurotus ostreatus* possessed a high reducing ability on the ferric cyanide complex, which also necessitated the recognition of the specimens as an antioxidant [31]. The further health effects of *Pleurotus ostreatus* could be attributed to its antioxidant activity, including the prevention and control of oxidative stress and cell damage and prevention of heart diseases through the regulation of cholesterol and inflammation and anticancer activity through the inhibition of cancer cell growth [32]. Also, these mushrooms exhibit antimicrobial, anti-inflammatory, and immunomodulatory properties [15]. However, more work must be carried out, especially involving human trials, to assert these noted health benefits as well as to determine how this works [33]. Therefore, *Pleurotus ostreatus* can be considered as a promising source of natural antioxidants due to the higher contents of phenolic compounds as well as essential and nonessential amino acids. It is important to note that the antioxidant content of oyster mushrooms and the associated health benefits should be utilized by consuming these mushrooms in our regular diets.

### 3.2. Mechanism of Action of Antioxidant Effect

It was established from several research reports that the bioactive compounds in oyster mushrooms act jointly to suppress oxidative stress and prevent the cells from being damaged by reactive oxygen species (ROS). The antioxidant properties are governed mainly by phenolic compounds, flavonoids, and ascorbic acid. The identified compounds, such as phenol, gallic acid, and ferulic acid, have pronounced radical scavenging characteristics, which reduce the free radical formation [34]. Moreover, like other mushrooms, the antioxidant capacity of this mushroom is also attributed to the presence of flavonoid antioxidants, which are important agents for antioxidation. Furthermore, this activity is further strengthened by the presence of ascorbic acid by scavenging the free radicals apart from protecting the body from oxidative stress [35]. The mechanisms through which *Pleurotus ostreatus* exerts its antioxidant effects include three processes as follows [36]: (a) The direct scavenging of free radicals—the antioxidant compounds present in the Pleurotus ostreatus can scavenge the free radicals, which halts the free radical chain reaction. This action assists in the protection of cells from oxidative damage to cellular structures, including lipids, proteins, and DNA. (b) The enhancement of antioxidant enzymes—*Pleurotus ostreatus* can enhance the activities of endogenous antioxidant enzymes, like superoxide dismutase, catalase, and glutathione peroxidase, and it strengthens the body’s immune system, assisting in the regulation of the levels of oxidants and antioxidants in the body, thus lowering the levels of stress. (c) The inhibition of free-radical-generating enzymes—this also suppresses the production or function of enzymes that create free radicals and, therefore, enhance its antioxidant properties.

## 4. Influence of Agricultural Practices, Extraction Method, and Storage Conditions on Composition of Bioactive Compounds of *Pleurotus ostreatus*

Regarding the studies conducted to find an ideal extraction method to optimize the antioxidant activity of the *Pleurotus ostreatus* extracts, it was reported that the extraction method used affects the composition of bioactive compounds extracted and, therefore, influences the antioxidant activity of the extracts. This outlines a process of enhancing the antioxidant activity by optimizing the extraction methods of the antioxidant amino acid, ergothioneine. Consequently, it has been found that acetone, ethanol, and water can all be used to extract antioxidants from *Pleurotus ostreatus*. Ethanol, especially at higher concentrations, seems to be the best solvent for the extraction of potent antioxidants from this mushroom [37]. As the extraction method has been reported to affect the composition of bioactive compounds of the extracts, there is a need to understand the influence of various solvents and extraction techniques concerning the extraction of antioxidants from oyster mushrooms before starting its large-scale production in food, medication, and beauty industries [38]. There are several quantitative analysis techniques for the determination of the antioxidant activity of *P. ostreatus*, namely DPPH radical scavenging activity and FRAP assay. It has also been found that the extracts of *P. ostreatus* are loaded with considerable antioxidant potential, but the potency is influenced by the extraction procedure and the solvent employed. For instance, it was observed that the ethanolic extracts have the highest antioxidant activity as compared to other solvent systems, meaning that the extraction methods can greatly affect the composition of the bioactive compounds. The study conducted in oyster mushroom *Pleurotus ostreatus* by Jayakumar et al. demonstrated that the ethanolic extract showed significant antioxidant activity, and this extraction method can be implemented in food as well as pharmaceutical industries. Furthermore, the in vitro study conducted by Rahimah and his colleagues also emphasized the same fact that ethanolic extract is the best for maintaining the antioxidant activity and reported that the antioxidant activity was higher in ethanolic extract than fresh and dry *Pleurotus ostreatus* mushroom samples [39,40]. Chukwu and colleagues researched the effect of different lime application rates on the yield and bioactive compound of two genotypes of oyster mushrooms. While this research was based on agricultural practices, the improvement of the conditions of growth can increase the content of bioactive compounds that have antioxidant and anticancer properties, which can be utilized for clinical purposes. Enhancing the yield and quality of oyster mushrooms creates more chances of using the mushrooms for therapeutic purposes, such as cancer prevention and treatment [20]. The stability of antioxidant properties of *Pleurotus ostreatus* depends on the conditions of storage, and a study on this showed that the antioxidant property of the mushroom is influenced by temperature, emphasizing the fact that the right storage condition should be maintained to utilize the maximum health benefits from the mushroom [41]. Oyster mushrooms stored at room temperature showed more antioxidant activity than frozen samples; hence, temperatures were found to influence antioxidant activity. Therefore, it is very important to note that the proper handling and storage at the right temperature maintains the health-promoting value of oyster mushrooms [30].

## 5. Oyster Mushroom Based Nutraceuticals

Mushrooms, the future super food that belongs to the fungi kingdom, are biologically distinct from the plant and animal kingdom, as they lack chlorophyll, but they can produce lignocellulolytic enzymes, and the mode of nutrition is saprophytic, parasitic, or symbiotic association with ligno-cellulolytic substrate. In general, “myco-nutraceuticals” are now creating innovative treatment methods as adjuvant therapy to fight against fatal diseases, like cancer and cardiovascular diseases, besides improving brain, heart, and gut health. Particularly, when comparing with other functional foods, like fruits, nuts, and vegetables, mushrooms are unique nutritionally due to the presence of essential amino acids, the vitamin D content, the higher protein content than fruits and vegetable, as well as the presence of medicinally important bioactive compounds [42]. Their umami flavor, fibrous texture, and high protein content make them an ideal alternative protein for meat analogue development as well as for incorporation in muscle foods [43,44,45,46,47,48]. In addition to the nutritional and medicinal benefits, *Pleurotus* species of mushroom production have also proved profitable from an economic point of view, as they have a wide range of substrate specificity and can be grown using several lignocellulosic agricultural wastes as substrate, which is sustainable as well as supporting the United Nation’s SDGs 1, 2, 3, 7, 9, and 12. Therefore, this production method of “wealth from waste technology”, using agricultural residues as lignocellulosic substrates, is cost effective, financially profitable, and environmentally friendly, mitigating the harmful greenhouse gas emissions, thus reducing the carbon footprint. Several researchers have optimized cultural parameters for the cost-effective production of oyster mushrooms. For instance, Lopez et al. successfully demonstrated the economic viability and sustainability of oyster mushroom production, utilizing waste from the coffee processing industry as substrate [49].

The active compounds isolated from these mushrooms are selective, which makes them suitable to be used in the development of nutraceuticals that provide health benefits that cannot be obtained from a normal diet [50]. Several clinical trials carried out by researchers and pharmaceutical firms have established that oyster mushroom extracts can enhance health and reduce the incidence of diseases by using them as functional foods [33]. The consumption of oyster mushrooms in nutraceuticals is based on their bioactive compounds that have the potential to prevent and reduce the severity of lethal diseases, such as cardiovascular diseases and cancer. These mushrooms are especially known for their antioxidant activity, which is vitally important for the body’s protection against oxidative stress [51]. Osteosarcoma (OSA) refers to a condition whereby the equilibrium between antioxidants and free radicals in the body is disturbed, a scenario that is common with aging or exposure to certain factors in the environment. This imbalance is a cause of cellular damage, premature aging, and disease, including cancer [37]. Oyster mushrooms have several anticarcinogenic compounds, including fiber, polysaccharides, polysaccharide–protein complexes, steroids, terpenoids, phenolics, and certain proteins. Some of the effects that have been associated with these compounds include the prevention of cancer growth, the improvement of the immune system, and the prevention of oxidant-induced injuries. For instance, polysaccharides, especially β-glucans, have immunomodulatory and antitumor properties, and phenolic compounds and terpenoids are antioxidants that will help in reducing free radicals and inflammation [52].

Other than the anticarcinogenic benefits, oyster mushrooms have several health benefits, including the improvement of the cardiovascular system, blood sugar regulation, and boosting the immune system [53]. Furthermore, mushrooms are rich in dietary fiber and assist in digestion and the management of cholesterol levels; the polysaccharides in these mushrooms have been associated with enhanced insulin sensitivity and glucose metabolism. All these potential health benefits have led to oyster mushrooms being commonly used in commercially produced nutraceutical items. Some of the commercially available oyster-mushroom-based nutraceuticals include Bulk Supplement’s Oyster Extract Powder, Organic Oyster Mushroom Powder, and Rooted Oyster Mushroom products [54]. These products are developed such that the active compounds are easily absorbed by the body, and therefore, the consumers can instantly obtain the therapeutic value of the oyster mushrooms. Also, oyster mushrooms are quite adaptable and can be eaten in forms, like powders, capsules, and extracts, thus offering consumers a convenient way to take them to increase their health status and to support against malnutrition and nutritional deficiencies [55]. Oyster mushrooms, as a part of the diet and as a food supplement, have the potential to play an important role in disease prevention and health enhancement, and this conforms with the current paradigm shift towards natural and complementary interventions [56]. Consequently, oyster-mushroom-based nutraceuticals are a breakthrough in functional food development. Thus, taking advantage of the natural pharmacological properties, these products can be useful in combating diseases, such as cancer and cardiovascular diseases, and in improving the general health of an individual [55]. Due to the ongoing research on the possibilities of these mushrooms, their use in the nutraceutical industry is also predicted to grow more and, thus, provide even more possibilities for the improvement of human health.

In summary, oyster mushrooms *Pleurotus* genus are one of the most known functional foods and attract the interest of researchers because of the presence of bioactive compounds and their positive effects on human health. Moreover, they are well known for their high nutrition content with a significant amount of protein content as well as with the presence of minerals, like iron and phosphorous, and vitamins, like riboflavin, thiamine, ergosterol, niacin, and ascorbic acid. Additionally, they are well recognized as a healthy food because of their low fat and energy content and by the presence of bioactive compounds, like terpenoids, flavonoids, like polyphenols, and polysaccharides, like glucans and glycoproteins. Furthermore, several preclinical studies have demonstrated that oyster mushrooms are hepatoprotective, immuno-modulating, anticancerous, antibacterial, antiviral, and hypo-cholesterolemic mediators. Moreover, their ability to prevent cardiovascular diseases and their potential to fight against cancer due to the presence of high antioxidant content, which reduces oxidative damage, make them an ideal candidate for nutraceutical production [57,58,59].

## 6. Manufacturing Process of Oyster Mushroom Nutraceuticals

**Figure 2:**
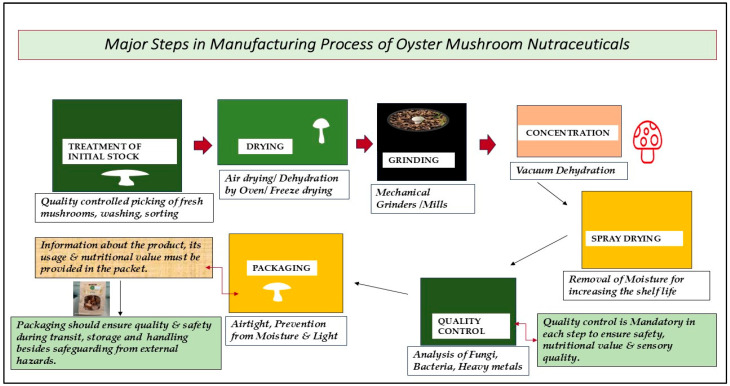
Diagrammatic illustration of the manufacturing process of oyster mushroom nutraceuticals.

According to recent trends, the major priority in food manufacturing and consumption is the functional components of the food. Functional ingredients in food are directly proportional to the antioxidant potential of the food. Based on recent research regarding *Pleurotus ostreatus* and the effect of substrate, it is clearly reported that the antioxidant capacity of *Pleurotus ostreatus* is influenced by the type of substrate [60]. Therefore, it is very important to optimize the culture conditions and physical parameters, especially the substrate constituents, before starting the large-scale cultivation of that specific mushroom for enhancing the maximum antioxidant potential. The procedure of manufacturing mushroom powder, especially for supplements, is quite precise and involves six important steps. The process involves first picking fresh and undamaged mushrooms, which are then washed and sorted out according to size. Quality control is very important at this initial treatment of stock stage, because the raw material, which is used in the production process, determines the quality of the final product [61]. The selected mushrooms are thoroughly washed to eliminate any form of dirt or any other unwanted particles. After cleaning the mushrooms, the mushrooms are dried, which serves to decrease the moisture level in them; this is important for avoiding spoilage and increasing the shelf life of the mushrooms. The drying process can be carried out using various methods, including air-drying, which is a physical process in which mushrooms are left to dry in the open air. The second drying method is dehydration using ovens or dehydrators at specific temperatures to dry the produce. Freeze-drying, another technique, is even more sophisticated, where the mushrooms are frozen and then dehydrated under vacuum to retain the nutrient value and taste [13]. Once dried, these are crushed or ground into fine particles using mechanical mills or grinders. This step is vital to ensure that the desired particle size distribution is attained, because this, in turn, will improve the solubility and, hence, the bioavailability of the active compounds in the final product [62]. Additionally, the mushrooms are concentrated by vacuum dehydration to remove excess water and then spray-dried to produce a fine powder. It is an effective way of maintaining the nutrient content and, at the same time, having a consistent product [63]. Quality control is an important aspect to be maintained throughout the stages of the production of foods. Finally, the oyster mushroom powder is analyzed for the presence of fungi and bacteria, as well as heavy metals, as well as analyzing the nutrient content. This ensures that the product is safe for use and is also effective, according to the nutritional and medicinal requirements. Finally, the oyster mushroom powder is packed in airtight containers to avoid moisture and light, which can otherwise affect the quality of the product. It should be noted that the presentation of the product, the information that is to be provided to the consumer about the product regarding its usage, and nutritional value also are very important. Mushroom powder for dietary supplement production is a multistep procedure that entails a lot of precautions at every stage, starting from the process of sourcing the right raw materials to the checking of the quality and standard of the final product. Particularly, each stage is as important as the other to ensure the quality and safety of the oyster mushroom nutraceutical product [64,65]. Figure 2 shows a diagrammatic illustration of the six major steps in the manufacturing process from fresh oyster mushrooms to dried oyster mushroom powder nutraceuticals.

## 7. Mechanisms of Action of Anticancer Activities of *Pleurotus ostreatus*

**Figure 3:**
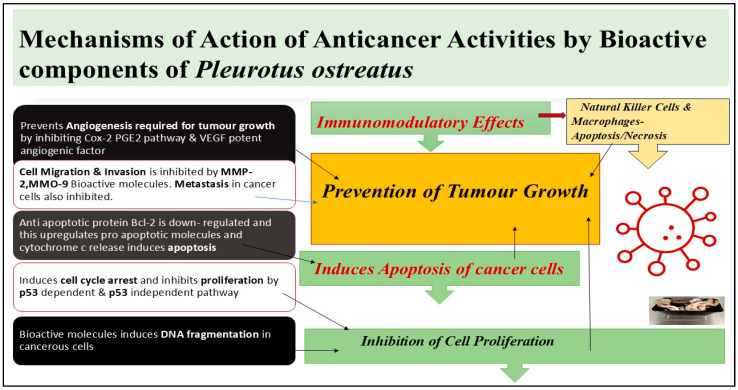
Mechanisms of action of anticancer activities by the bioactive components from *Pleurotus ostreatus*.

The major way by which *Pleurotus ostreatus* has been suggested to exert its anticancer activities is through the suppression of cancer cell growth, and it has been established that the extracts can prevent the growth of breast cancer cell lines, namely MCF-7 and MDA-MB-231, as well as colon cancer cell lines HT-29 and HCT-116. Growth arrest is usually linked with cell cycle arrest, and therefore, the inhibition of cell proliferation is usually followed by cell cycle arrest [66]. Research shows that *Pleurotus ostreatus* extract can cause cancer cells to remain in the G0/G1 phase of the cell cycle and, thus, stop dividing. This effect is mediated through the increased expression of tumor suppressor proteins, such as p53 and p21, which are involved in cell cycle regulation and apoptosis, respectively. Also, the regulation of retinoblastoma (Rb) protein phosphorylation is important for the preservation of this cell cycle arrest [54]. Another important method of cancer suppression action is the initiation of apoptosis, or programmed cell death, in cancer cells. *Pleurotus ostreatus* has several bioactive molecules in its body, including polysaccharides, like β-glucans, and these have been known to activate apoptosis. For instance, it has been found that polysaccharide extracts from *Pleurotus ostreatus* can cause cancer cell death in colon cancer through a process known as apoptosis, which is regulated by caspases. These activated caspases cause the cleavage of some cellular components and the final death of the cancer cells. Moreover, the extracts of mushrooms can alter the levels of the proteins that promote cell death through apoptosis and those that oppose it, favoring the former. *Pleurotus ostreatus* has also been reported to possess immunomodulatory effects that can also contribute to the anticancer properties of the mushroom. It has been established that the mushroom has immunomodulating effects; therefore, it increases the activity of natural killer (NK) cells and macrophages [53]. These immune cells are very important in the identification and elimination of cancer cells and a diagrammatic illustration of the mechanism of action of anticancer activities of bioactive molecules present in *Pleurotus ostreatus* is shown in Figure 3. Studies have shown that compounds isolated from *Pleurotus ostreatus* can enhance the production of cytokines IL-2 and TNF-α that are useful in the action against tumor cells. Thus, *Pleurotus ostreatus* can enhance the immune system’s activity against cancerous cells to eventually prevent tumor growth [67]. Bioactive compounds, like phenolic compounds and β-glucans, have immune-modulatory activity and enhance cellular components of the immune system. β-glucans bind to innate immune cells through pattern recognition receptors (PPRs), leading to a cascade of signaling pathways, ultimately results in activation of cells. Oyster mushroom glucans are then engulfed by macrophages, which are then fragmented. Fragmented glucans released from cells activate natural killer (NK) cells and granulocytes, which in turn release perforins and granzymes that lead to the disintegration of the DNA of tumor cells. In fact, the activated granulocytes are responsible for the protection of the body from pathogens and the activated macrophages release cytokines and interferons that lead to the activation of cytotoxic T lymphocytes (Tc) as well as helper T lymphocytes (Th), which are further responsible for the immunomodulatory action against pathogens [50].

## 8. Anticancer Studies of *Pleurotus ostreatus* in Different Cancer Cell Lines—In Vitro Studies

Various investigations have revealed that *Pleurotus ostreatus* has potential in the treatment of breast cancer, the most invasive cancer found in women globally. For instance, some compounds isolated from the mushroom have been seen to suppress the movement and penetration of breast cancer cells, which are among the vital steps in the development of metastases. The apoptosis of breast cancer cell lines adds to the evidence as to why it can be used as a therapeutic agent. It has also been observed that *Pleurotus ostreatus* has good anticancer activity against colon cancer cells. It has been discovered that polysaccharides from the aqueous extract of oyster mushrooms suppresses the proliferation of HT-29 colon cancer cells and triggers apoptosis. Because of their action on cancer cells to decrease their viability, this specific bioactive compound can be suggested to be used as a dietary supplement for the prevention of cancer in clinical trials [23]. Polysaccharide POMP-2 (Pleurotus *ostreatus* mycelium polysaccharide-2) with a molecular weight of 29 kDa extracted from *Pleurotus ostreatus* mycelium showed antitumor activities on gastric cancer both on in vitro cell lines as well as in an in vivo experiment conducted in mice [68]. The most common type of liver cancer, hepatocellular carcinoma (HCC), has been a focus of the most recent studies on *Pleurotus ostreatus*. Previous studies have also pointed out that certain compounds of the mushroom can inhibit HCC cell proliferation and induce apoptosis and, thus, limit the growth of the tumor. The polysaccharides in *Pleurotus ostreatus* have been linked with these effects and, thus, may have potential for the development of new therapeutic measures against liver cancer [36]. Therefore, *Pleurotus ostreatus* has antitumor effects in multiple ways, such as the suppression of cell growth, the promotion of apoptosis, and boosting immunity. This makes it a promising candidate for use as a natural therapeutic agent in cancer prevention and treatment plans against breast, colon, or hepatocellular cancer. Another study regarding extracts from *Pleurotus ostreatus* reported that molecular docking can be utilized to find the cytotoxic activity of specific compounds in the extract and methyl gallate in the extract has a cytotoxic effect and, therefore, has a significant role against breast cancer cell line MCF7 [69]. Although the results of in vitro and animal studies are encouraging, further research, especially clinical trials, is needed to fully understand the action of *Pleurotus ostreatus* to ensure effectiveness in the treatment of cancer in humans. Thus, the obtained results indicate the possibility of the use of *Pleurotus ostreatus* as a source of bioactive compounds with anticancer properties for further future studies and potential applications. Other in vitro studies conducted regarding the anticancer effects of different types of bioactive compounds extracted from *Pleurotus species* on cancer cell lines are illustrated in Table 1.

## 9. Anticancer Effect of Different Species of Oyster Mushrooms—In Vivo Studies

Several animal studies regarding the anticancer properties of *Pleurotus* species have been conducted and observed that the extracts of specific bioactive compounds as well as fresh or dry oyster mushrooms demonstrate significant anticancer activity against breast cancer, colorectal cancer, cervical cancer, hepatocellular cancer, and blood cancer. Krishnamoorthy et al. demonstrated that ethanol extract of *Pleurotus ostreatus* has a positive impact on the prevention of DMBA (7,12-Dimethyl benzanthracene)-induced mammary carcinogenesis in female rats. The oral administration of the extract resulted in a significant decrease in tumor volume as well as enhancement in the body weight of the animals, without changing their regular diet and water intake. It was also concluded that a higher dose of the extract rather than a lower dose reduced breast cancer by inducing the cytotoxicity of the specific cells by the apoptotic pathway, particularly due to the presence of ergosterol [74]. Similarly, in a research study conducted by Ida et al. reported that beta-glucan extracted from *Pleurotus ostreatus* fed to DMBA-induced rats with breast cancer resulted in a reduction in tumor volume as well as a reduction in the total number of tumor nodules [75]. Table 2 shows a detailed illustration of the other reported in vivo studies conducted regarding the anticancer effects of different species of oyster mushrooms.

## 10. Clinical Studies and Trials Regarding Oyster Mushrooms

Several clinical studies and trials have been carried out with oyster mushroom and its supplements to support the research reports based on their antioxidant activity, bioactive compounds, and to prove how they can be beneficial for human health. In this section, information regarding the main clinical trials that have investigated the therapeutic possibilities of oyster mushrooms concerning several clinical ailments is presented. Research carried out by Abrams and his colleagues aimed at identifying the antihyperlipidemic properties of *Pleurotus ostreatus* among HIV-positive patients. Nonetheless, the study also pointed out that the lipid-lowering effects of oyster mushrooms were also associated with their antioxidant properties. All the participants were already at an increased risk of oxidative stress due to their HIV status and their receipt of antiretroviral therapy (ART); they benefited from consuming oyster mushroom supplements in terms of improved lipid profiles. The present study indicates that the antioxidant properties of *Pleurotus ostreatus* may be responsible for the lipid-lowering effects that may help in decreasing the risk of cancer among this population [72]. In another cross-sectional study, Uffelman and colleagues chose to focus on the effects of mushroom consumption on cardiometabolic risk factors in a Mediterranean-like diet and reported the antioxidant capacities of the mushrooms, including oyster mushrooms. The research revealed that the short-term intervention of regular mushroom consumption did not have a material impact on most of the cardiometabolic risk factors. Nevertheless, the antioxidant activity of mushrooms may still have some long-term health effects, such as cancer prevention when consumed as a balanced diet [73]. Tian and colleagues analyzed how the human gut mycobiome changed in a day and over several days, in response to diets with different macronutrient ratios. This work did not concentrate on oyster mushrooms alone but provided insights into the effect of a diet on mycobiome, which is relevant to the immune system and health. Consequently, the results indicate that consuming mushrooms, including *Pleurotus* species, can alter the gut mycobiome and, therefore, improve the body’s prevention against cancer through the enhancement of gut health and immune control [84].

Several studies have pointed out that there are some limitations regarding the reduced bioavailability of some bioactive compounds, like polyphenols, from oyster mushrooms when taken orally, since this will be recognized as xenobiotics, leading to its poor absorption level in the intestine. The definition of bioavailability in the nutritional viewpoint when carrying out preclinical or clinical studies is the quantity of bioactive compounds absorbed and that can be utilized for the requirement of the animal or human body [85]. The bioavailability of mushroom β-glucan and flavonoids is an important research question extensively studied, and many studies are ongoing regarding the immune modulating potential to develop mushrooms as a nutraceutical for adjuvant therapies against fatal diseases, like cancer. The β-glucan extract from *Pleurotus* species of mushrooms is named “pleuran”. As the preclinical studies regarding β-glucans showed positive anticancer results in animal models, clinical trials in cancer patients were conducted, in combination with chemotherapy, and it was observed that β-glucan extracts from oyster mushrooms can be successfully used as a complementary cancer therapy for gastrointestinal cancer patients. Similarly, as β-glucans showed antitumor effects, clinical trials are now ongoing regarding the usage of β-glucan adjuvant therapy along with monoclonal-antibody-mediated immunotherapy, which can pave the way for the latest innovation in cancer treatment [86,87,88]. Flavonoids, which are a family of polyphenolic compounds, are also extracted from oyster mushrooms and have shown strong anticancer potential due to the presence of antioxidants that help to neutralize reactive oxygen species (ROS), thus protecting the cells from oxidative stress. Furthermore, polyphenols influence multiple immunomodulatory processes involved in the inflammatory responses and carcinogenesis, which can be applied to the treatment of immunocompromised patients with malignancies [89].

Kleftaki and his coresearchers carried out clinical trials on the impacts of *Pleurotus eryngii* on clients with metabolically untenable obesity. The study established that the intake of this mushroom species reduced postprandial glycemic and suppressed ghrelin, the hormone that regulates hunger. These results indicate that the *Pleurotus* species can not only help in the regulation of metabolism but may also help in cancer prevention by decreasing the obesity-induced cancers. This study reinforces the consumption of oyster mushrooms for their versatility in the improvement of health and the prevention of diseases, such as cancer, through the promotion of better metabolism [90]. Preclinical trials or studies in animal models help researchers and clinicians to assure the safety of the bioactive compounds extracted as well as the safety of fresh mushroom or dry mushroom powder in the form of nutraceuticals before moving into the clinical trials with cancer patients. Additionally, the studies also confirm the approximate dose or how much mushroom quantity should be given at specific time intervals. In fact, several secondary metabolites with anticancer bioactivity extracted from oyster mushrooms reported by preclinical studies are under clinical trials now, with the expectation for obtaining their approval as an adjuvant for complementary cancer therapy.

Apart from oyster mushrooms, several other mushrooms belonging to different genus and species have been studied to evaluate their anticancer potential, and it should be noted that only the ones with the most potential will receive approval for clinical trials. In the review article by Lam et al., they specifically detailed the herb–drug interaction studies of medicinal mushrooms. Lingzhi and Yunzhi clearly emphasized that cytotoxic drugs during clinical trials showed better survival and improved the quality of life of cancer patients, reduced tumor lesions, immune modulation, and the mitigation of chemotherapy side effects [91]. Polysaccharides extracted from *Grifola frondosa* and *Trametes versicolor* gave a positive result for breast cancer treatment and successfully finished the phase 1 clinical trial. The semisynthetic anticancer drug illudin-S purified from the mushroom *Omphalotus* genus showed effective results against prostate, ovarian, and renal cell carcinoma, and it was established that their mode of action is through DNA replication. Polysaccharide lentinan isolated from *Lentinula edodes* when used as a complementary cancer therapy showed considerable improvement in the quality of life of cancer patients as well as positive treatment results for lung, gastric, colorectal, and uterus cancers. Other mushroom species successfully evaluated for anticancer clinical trials were *Ganoderma lucidum* and *Coriolus versicolor,* while different species of *Agaricus* mushrooms are also now undergoing clinical trials for the evaluation of different types of anticancer treatments [92].

Regarding the preclinical studies on the quantity of anticancer bioactive compounds to be administered, results were obtained from the in vivo studies on female mice; it was established that 1 g *Pleurotus ferulae* ethanol extract (PFEC) per kg of body weight is an effective dose for the treatment of skin cancer. Similarly, another preclinical study conducted on female mice emphasized that 20–40 mg of *Pleurotus ferulae* Ergosterol peroxide (PFEP) per kg of the body weight is an effective dose for the treatment of gastro-intestinal cancer in conjunction with chemotherapy. It was also established that rather than fresh mushroom intake, extracts and powdered forms of nutraceuticals are effective for complementary cancer therapy [93]. Meanwhile, by conducting a double-blind placebo-controlled clinical trial, Tanaka et al. established that oyster mushrooms, with common name Tamogitake, have anticancer potential through the upregulation of the immune system. The oral administration of the oyster mushroom extract for 8 weeks increased interferon IFN and IL-12, besides a small increase in NK activity [94]. A clinical trial conducted on cervical and gastric cancer patients at stage II or III by the administration of oyster extract from *Pleurotus cornucopiae* showed a reduction in tumor size [92]. Several clinical trials are ongoing based on previous preclinical studies, and scientists and clinicians are working for the further development of this “nutra-mycoceutical” as a potential anticancer dietary supplement that could be exploited in complementary cancer therapy.

## 11. Challenges in Utilizing Therapeutic Benefits of Oyster Mushrooms

While the potential of oyster mushrooms as a complementary therapy in cancer treatment is promising, several challenges must be addressed to fully utilize their therapeutic benefits. This section discusses the key challenges in current research. Variability in bioactive compound content has been seen in the species, according to the conditions under which the oyster mushrooms are grown and the substrates used because the environmental factors greatly influence the concentration and composition of bioactive compounds present in these mushrooms. This variability is a problem in the standardization of the therapeutic potency of oyster mushrooms, which makes it hard to establish the right dose and form of the mushrooms to use in cancer treatment. The lack of a standard bioactive compound profile could, therefore, weaken the reliability and effectiveness of the oyster-mushroom-based treatments [40]. The lack of large-scale clinical trials is another challenge in utilizing the therapeutic benefits of oyster mushrooms. Although many preclinical and small-scale randomized controlled trials have proved the anticancer and antioxidant effects of oyster mushrooms, there is limited evidence regarding their efficacy in different cancer patients due to lack of large-scale clinical trial evaluation. Moreover, it is difficult to determine the effectiveness and safety of oyster mushrooms in cancer treatment and management, as it is not easily accepted by the medical fraternity. Furthermore, the potential interaction of oyster mushroom bioactive compounds with conventional cancer medicines is not well known. The effect of oyster mushroom intake in the regular diet of the cancer patients and its synergic effect with standard cancer treatment protocols, including chemotherapy and radiation, must be studied to establish the effect of oyster mushrooms on the efficacy of anticancer treatments and the possible side effects due to interactions and contradictions. It is important to note that the knowledge of these interactions is vital in the incorporation of oyster mushrooms in the current cancer treatment regimens [95]. Consumer accessibility and perception are also very important; although, oyster mushrooms are becoming a popular functional food for cultivation and consumption across the globe. The availability and acceptance of this as a dietary supplement are not uniform across the world, and cultural reasons, accessibility, price, and awareness of the consumer are other challenges preventing the use of oyster mushrooms for health improvement purpose [28]. Moreover, mushrooms are not common in areas where they are not part of the diet culture; this may be due to a lack of knowledge regarding their health benefits, and unawareness about their medicinal importance and their application in cancer prevention and treatment is being limited. There are still issues with the regulation and standardization of mushroom-based supplements, especially relating to product quality, safety, and efficacy. On the contrary, there is no regulation on the production, processing, and packaging of oyster mushrooms, and hence, the quality of the product may not fulfil the nutritional and medicinal requirement standards [96]. Furthermore, there are problems regarding the standardization of the effective dose and the lack of clinical trials, which is the major challenge for the integration of oyster mushroom products into healthcare delivery systems as therapeutic agents.

## 12. Future Directions to Overcome the Challenges

Here, we discuss the application and outlines of future directions to overcome the existing challenges for the utilization of oyster mushrooms in cancer therapy.

### 12.1. Standardization of Cultivation and Extraction Processes

Further studies should be directed towards the optimization of the cultivation parameters and extraction conditions for oyster mushrooms to achieve uniform levels of bioactive compounds. Some of the strategies that can be employed include the control of growing conditions, substrate, and postharvest management to enhance the levels of bioactive compounds, such as polysaccharides and phenolic compounds, which are important for the standardization of oyster-mushroom-based therapies for anticancer treatment.

### 12.2. Conducting Large-Scale Clinical Trials

To confirm the effectiveness of oyster mushrooms in cancer treatment, large randomized controlled clinical trials that include an extensive number of patients should be carried out. These trials should also establish the side effects of consuming oyster mushrooms in the long run, the right quantity to consume, and their effectiveness in treating the various types of cancers. Such studies would offer sound clinical data that may be used to justify the use of oyster mushrooms in standard cancer therapy.

### 12.3. Investigating Synergistic Effects with Conventional Therapies

Further research should be conducted regarding the effect of the interaction of oyster mushroom bioactive components with conventional cancer therapy. This involves understanding the molecular processes of these interactions and determining if oyster mushrooms can increase the efficacy of chemotherapy, radiotherapy, or immunotherapy. This could provide the basis for the design of more efficient complementary cancer therapy.

### 12.4. Enhancing Consumer Education and Accessibility

Measures should be taken to promote the consumption of oyster mushrooms, because they are not widely known and consumed in some countries. Health awareness programs, educational interventions, and collaboration with local farmers on the growth of oyster mushrooms should be encouraged. Also, sufficient steps should be taken by the authorities to make oyster mushrooms easily available in the market, at a reasonable price.

### 12.5. Development of Mushroom-Based Nutraceuticals

The advancement of nutraceuticals derived from oyster mushrooms as an anticancer preparation is a highly innovative development in future cancer therapy. These products must be formulated to contain high levels of bioactive compounds in a convenient, pharmacological form, for example capsules or extracts. The researchers, healthcare providers, and the nutraceutical industry will need to work together for this innovation. It is also important to note that certain rules and regulations must be established regarding the utilization of oyster mushroom nutraceuticals to broaden their applicability in the existing healthcare system.

### 12.6. Exploring New Applications and Species

Besides *Pleurotus ostreatus*, other oyster mushrooms, like *Pleurotus eryngii* and *Pleurotus pulmonarius*, also have bioactive compounds that can be useful in cancer treatment. Further studies should be carried out on these species and other rare mushrooms to determine the other possibilities of their uses in cancer prevention and cure. Oyster mushrooms are a possible new source of complementary cancer treatment, as they have antioxidant, immunomodulatory, and metabolic effects. Therefore, the current challenges of standardization, clinical validation, and regulatory approval as therapeutic agents should be overlooked. By following the future directions mentioned above, researchers and healthcare professionals can enhance the application of oyster mushrooms in the treatment of cancer and, thus, the overall welfare of the patients as well as establishing the position of natural products in contemporary healthcare systems.

### 12.7. Development of Colloidal System and Nanosystem

The reduced bioavailability of some bioactive compounds, like polyphenols from oyster mushrooms when taken orally, is due to its recognition as a xenobiotic, leading to its poor absorption level in the intestine. To overcome this drawback, researchers are now focusing on the development of colloidal systems as well as utilizing nano systems [50,65].

### 12.8. Oyster Mushroom Production in Controlled Conditions with Standardized Substrates

Another major drawback regarding the commercialization of such bioactive components is variation both qualitatively and quantitatively due to differences in locality and the environmental conditions of growth, as they can absorb these bioactive components from the substrate. The solution for reducing this variability is by growing oyster mushrooms in controlled conditions, under the optimized conditions of the substrate concentration and other physical parameters [50,65].

### 12.9. More Clinical Trials to Overcome Adverse Effects Like Interaction with Other Treatments

Mushroom nutraceutical–drug interactions may lead to either beneficial or harmful health effects in cancer patients. Therefore, further studies regarding the interaction between oyster mushroom bioactive compounds and chemotherapy drugs should be evaluated through preclinical trials in animal models as well as with long-term, double-blind, and placebo-controlled clinical trials on an extensive number of cancer patients [91].

## 13. Concluding Remarks and Future Prospects

In general, all these works confirm the high potential of oyster mushrooms in adjuvant cancer treatment. The components of oyster mushrooms include polysaccharides and phenolics as well as ergosterol derivatives. In fact, all these bioactive compounds that were extracted and purified have been known to have high antioxidant activity, which is essential in the elimination of free radicals and the prevention of oxidative damage to cells that are one of the leading causes of cancer [20]. Among the multiple therapeutic effects of oyster mushrooms, the potential to regulate the immune response is another important factor. Oyster mushrooms can, thus, be used as a complementary therapy along with the conventional cancer treatments, which would boost the immune system as well as eliminate the cancer cells. For instance, the immunomodulatory properties of beta-glucans present in *Pleurotus* species have been well studied and described in publications and could be utilized for the prevention and treatment of cancer [20]. Also, the effects of oyster mushrooms on metabolic health, particularly regarding obesity and glycemic control, which are leading factors in cancer development, are of significant interest, while incorporating oyster mushrooms in diets could be useful for protecting the body from these cancer risks.

This review provides insight into the potential of oyster mushrooms as functional foods in cancer prevention and management as well as in improving overall health conditions, since oyster mushrooms have been a promising sustainable source of bioactive compounds. Their proapoptotic effect on cancer cells and antitumor properties makes them a good candidate for acting as an adjuvant in cancer treatment. While the current studies are promising, more studies are needed to identify the compounds that are responsible for these effects and to prove the effectiveness of these compounds in human clinical trials. It is important to note that, due to the increasing global incidence of cancer, the consumption of oyster mushrooms as a part of the regular diet is a necessary, feasible method of utilizing nutraceuticals to improve people’s health globally. It should be noted that laboratory research studies and preclinical studies in animal models are important before going for clinical studies regarding nutraceutical-based, complementary cancer therapy. It is expected that knowledge and awareness regarding the health qualities of oyster mushrooms through a greater number of clinical trials may open new frontiers of nutritional interventions for boosting the survival and wellbeing of cancer patients. It is, therefore, recommended that further research is conducted by the scientific fraternity on these oyster mushrooms, not just for the nutritional security of the world but for their ability to combat cancer and other forms of fatal diseases. To overcome the obstacles of the application of oyster mushroom nutraceuticals in formal medical therapies, more precise clinical studies must be conducted. In conclusion, oyster mushrooms must be considered as one of the most promising natural resources in the battle against cancer and as antioxidants, immunomodulators, and modulators of metabolic health; they are potentially useful as an adjunct in cancer prevention and control, when utilized along with other current cancer treatment methods. Nevertheless, not many clinical trials have been conducted regarding the *Pleurotus* genus of mushrooms. It is also very important to study bioactive compound–chemotherapy drug interaction through preclinical studies as well as with an extensive number of clinical trials in cancer patients to fully utilize this gift from nature for oncology treatment.

## Figures and Tables

**Table 1:** Anticancer studies on different types of cancer cell lines by different species of oyster mushrooms.

Mushroom Species	Cancer Cell Line Types	Anticancer Effect of Bioactive Compounds	References
*Pleurotus ostreatus*	Breast Cancer (MCF-7)	Polysaccharides induce antiproliferative and proapoptotic effects, through p53 dependent and p-53 independent pathways and enhanced immune response. Aqueous polysaccharides inhibit proliferation through p53 dependent and p-53 independent pathways and induce apoptosis. Anthraquinone induces apoptosis and inhibits MMP-2 and MMP-9 expression in MCF-7 cells. Phenolic compounds exhibit cytotoxic effects and induce apoptosis in breast cancer cells. Bioactive compounds show antiproliferative effects on colon cancer cells. β-glucan exhibits antiproliferative effects and enhances immune responses. Polysaccharides show significant antitumor activity against lymphoma cells. Polysaccharides induce antiproliferative and proapoptotic effects. Ethanol extracts exhibit antioxidant, anti-inflammatory, and antitumor properties. Ergothioneine acts as an antioxidant, potentially reducing oxidative stress and lowering cancer risk. Anthraquinone induces apoptosis via the mitochondrial pathway, inhibits MMP-2 and MMP-9 expression, and reduces cell viability. β-glucan exhibits antiproliferative effects, induces apoptosis, and modulates immune responses to enhance anticancer activity. Polysaccharides induce antiproliferative and proapoptotic effects on HT-29 colon cancer cells, enhancing immune response. Ergothioneine acts as an antioxidant, reduces oxidative stress, and lowers cancer risk. Extracted fractions induce apoptosis and reduces cell viability in breast cancer cells.	[70]
*Pleurotus ostreatus* *Pleurotus ostreatus*	Colon Cancer (HT-29) Breast Cancer		[34] [70]
*Pleurotus jammer*	Breast Cancer (MCF-7)		[71]
*Pleurotus citrinopileatus*	Colon Cancer (HCT-116)		[12]
*Pleurotus ostreatus*	Breast Cancer		[34]
*Hypsizygus ulmarius*	Lymphoma		[12]
*Pleurotus ostreatus*	Colon Cancer (HT-29)		[34]
*Hypsizygus ulmarius*	Breast Cancer (MCF-7)		[12]
*Pleurotus ostreatus*	Various Cancer Cell Lines		[16]
*Pleurotus ostreatus*	Breast Cancer		[72]
*Pleurotus ostreatus*	Breast Cancer		[72]
*Pleurotus ostreatus*	Colon Cancer		[73]
*Pleurotus ostreatus*	Various Cancer Cell Lines		[73]
*Pleurotus highking*	Breast Cancer (MCF-7)		[36]

**Table 2:** In vivo anticancer activity studies of different species of oyster mushrooms on animal models.

Mushroom Species	Animal Models	Anti-Cancer Effect	References
*Pleurotus ostreatus*	Colon cancer cell bearing male Wistar rats	Pleuran reduced precancerous aberrant crypt foci lesions in colon. Reduced expression of cyclogenase-2, K1-67, Cyclin D1, and F4/80. Flavanoid content of ethanol extract increased the survivability rate of mice and decreased the tumor volume. Alkali extracted polysaccharide PNA2, inhibited the migration of Hep G2 cells and suppressed tumor growth. Metabolites from both *Pleurotus* species increased hemoglobin concentration and anti-anemic effect, prevented leukocytosis, increased total leukocytic content. Water soluble proteoglycan enhanced cytotoxicity of NK cells and stimulated macrophages to release nitric oxide in a dose dependent manner. Recombinant ostreolysin reduced tumour growth. Polysaccharides showed tumour suppression against hepatic cellular carcinoma. Polysaccharides reduced tumour size and mass, and organ toxicity. Moreover, showed improved health and longevity.	[76]
*Pleurotus ostreatus* *Pleurotus ferulae*	Colon Cancer induced mice Hepato cellular Tumor bearing male mice		[77] [78]
*Pleurotus* *nebrodensis*	Human hepatic cancer cell (Hep G2) bearing mice		[79]
*Pleurotus ostreatus, Pleurotus pulmonaris*	Leukemia bearing Wistar rats		[80]
*Pleurotus ostreatus*	Sarcoma 180 bearing mice		[81]
*Pleurotus ostreatus*	Mice xenografted with colon cancer		[6]
*Pleurotus ostreatus*	Mice models induced with hepatic H22 carcinoma		[82]
*Pleurotus ostreatus*	Ehrlich Ascite Carcinoma (EAC) bearing Swiss Albino Mice		[83]

## Data Availability

No new data were created or analyzed in this study. Data sharing is not applicable to this article.
